# Editorial: Neuropsychology and Neuropsychiatry of Neurodegenerative Disorders

**DOI:** 10.3389/fnagi.2015.00227

**Published:** 2015-12-16

**Authors:** Manuel Menéndez-González, Tania Álvarez-Avellón

**Affiliations:** ^1^Unidad de Neurología, Hospital Álvarez-BuyllaMieres, Spain; ^2^Departamento de Morfología y Biología Celular, Universidad de OviedoOviedo, Spain; ^3^Instituto de Neurociencias del Principado de Asturias, Universidad de OviedoOviedo, Spain; ^4^Facultad de Ciencias de la Salud, Universidad Autónoma de ChileTalca, Chile; ^5^Departamento de Psicología, Universidad de OviedoOviedo, Spain; ^6^Neuropsicología, VitalAsturGijón, Spain

**Keywords:** neuropsychology, neuropsychiatry, neurodegenerative diseases, mild cognitive impairment, parkinson disease, alzheimer disease, neuroimaging, neuropsychological tests

This Research Topic is published to gather some of the latest science around neuropsychology and neuropsychiatry in neurodegenerative disorders.The call was launched in 2014 and 20 articles were eventually accepted and published, including papers on language and visuospatial functions, emotion, psychometry, brain morphometry, brain connectivity, diagnostic tests, and interventional studies in different conditions, mostly Alzheimer's disease (AD) and Parkinson's disease (PD). Interestingly, several articles focused in the early stages of these diseases. This paper you are reading is the editorial article introducing these publications.

Let's start by the opinion article by Laurent and Noiret, elaborating on visual-motor embodiment of language and the implications for the neuropsychological evaluation in AD (Laurent and Noiret, 2015). They remind us how much cognition is situated, grounded and embodied in specific perceptual and perceptual-motor systems, which allow recursive processes, conceptual elaboration, and the enaction of modular “cognitive functions.”

In a general commentary article, Moustafa commented on the paper “Effects of aging and involuntary capture of attention on event-related potentials associated with the processing of and the response to a target stimulus” by Cid-Fernández et al. (2014). Moustafa says Cid-Fernandez et al. findings have implications for the understanding of motor and cognitive problems associated with age-related neurodegenerative disorders, including PD and AD (Moustafa, 2014).

We are happy to say 17 original articles were published in this Research Topic. We want to start highlighting the one by Drummond et al., who showed deficits in narrative discourse elicited by visual stimuli are already present in patients with mild cognitive impairment (Drummond et al., 2015). This study evaluated parameters for investigating narrative discourse in patients with AD and amnestic Mild Cognitive Impairment (a-MCI) and a control group. The Control and AD groups differed in all parameters except narrative time and the total number of words recalled. The a-MCI group displayed mild discursive difficulties that were characterized as an intermediate stage between the Control and AD groups' performances. The a-MCI and AD groups were similar to one another but differed from the control group with respect to the type of words recalled, the repetition of words in the same sentence, the narrative structure and the inclusion of irrelevant propositions in the narrative. The narrative parameter that best distinguishes the three groups was the speech effectiveness index.

Continuing with visual stimuli, Yin et al., assessed the visuospatial characteristics of an elderly Chinese population using the Wechsler Adult Intelligence Scale-Revised (WAIS-R) Block Design Test (BDT) (Yin et al., 2015). They found that simple BDT task scores can distinguish demented patients from MCI, while difficult BDT task scores can ease discriminating between controls and MCI. Thus, normative data stratified by education and age for the Chinese elderly populations are provided to be used in the diagnosis of dementia and MCI in this population.

More on psychometric scales and populations; researchers from Brazil showed the psychometric properties of the Brazilian version of the FAQ (P-FAQ) has god ecological validity, reliability, internal consistency and construct validity. Therefore, this questionnaire is now ready to be used in the Brazilian population of older adults (Assis et al., 2014).

From psychometric to neuroimaging studies: Balardin et al., studied the differences in prefrontal cortex activation and deactivation in MCI using fMRI while encoding word lists (Balardin et al., 2015). MCI individuals showed reduced free recall scores when using self-initiated encoding strategies but they were increased to baseline controls' level after receiving directed instructions. Greater recruitment of front parietal regions was observed in both MCI and control groups during directed strategic encoding. In conclusion, this study provides evidence showing that differences of activity in these regions may be related to encoding deficits in MCI, possibly mediating executive functions during task performance.

Authors from Japan, studied how variations in WMH volume and cortical thickness relate to episodic memory, depressive state, and the presence of MCI (Fujishima et al., 2014). MCI participants exhibited thinner cortices in the inferior parietal and temporal lobes and greater WMH volumes in the semioval center and corona radiata than controls. Also, poor episodic memory was associated with increased WMH volume in the posterior periventricular regions and thinner cortices in the left entorhinal region in MCI participants. Compared with non-depressed MCI participants, depressed MCI participants showed greater WMH volume as well as reduced cortical thickness in the gyrus adjacent to the amygdala and in the anterior medial temporal lobe bilaterally. In MCI participants, a higher WMH volume was associated with cortical thinning in the frontal, temporal, and parietal regions. In conclusion, these results confirm that “depression and episodic memory are associated with both cortical thickness and WMH volume in MCI subjects.”

A group of authors from California (USA), studied the interactive effects of vascular risk burden and advanced age on cerebral blood flow (Bangen et al., 2014). Authors conclude that “older adults with elevated vascular risk burden may be particularly vulnerable to cognitive change as a function of CBF reductions.” Then CBF might be used as a potential biomarker in preclinical AD.

Graph theory is a mathematical approach to analyze relations between items and represents a promising tool to understand neuropsychological states. Graph analysis is even likely to become clinically relevant in neurology and psychiatry, being particularly useful for the differential diagnosis of different conditions. A couple of articles in this Research Topic used graph analyses. A group of authors from Brazil showed graph analysis of verbal fluency test discriminate between patients with AD, mild cognitive impairment and normal elderly controls (Bertola et al., 2014a). This research provides support for a new methodological frame to assess the strength of semantic memory through the verbal fluency task, with potential to amplify the predictive power of this test. The same group of authors also showed in a different article how impaired generation of new subcategories and switching is in a semantic verbal fluency test in older adults with mild cognitive impairment (Bertola et al., 2014b). This finding indicates that semantic memory impairment is a visible and recent deficit that occurs even in non-demented subjects with MCI.

It is well known that cognitive decline and dementia due to AD are associated with lifestyle, genetic and environmental factors. Several potentially modifiable risk factors should be considered for preventive or ameliorative interventions in AD. The list of such risk factors is still expanding. Researchers from Australia found bone mineral density (BMD) and body composition are two of such potentially modifiable risk factors (Sohrabi et al., 2015). Specifically, researchers found the List A learning from California Verbal Learning Test was significantly associated with lean mass and BMD. These findings indicate that “there is an association between BMD and lean body mass and episodic verbal learning.”

Continuing with studies addressing the impact of cognition and emotional status on daily functioning, de Paula et al., cols investigated if depressive symptoms and specific cognitive domains affect different aspects of activities of daily living (ADL) (de Paula et al., 2015). They observed that depressive symptoms were predictive of ADL involving social contact and that different instrumental ADL have specific cognitive predictors. Authors conclude that there are specific patterns of influence depending on the specific instrumental ADL.

Iturria-Medina and Evans, from Canada, reviewed the role of brain connectivity in the progression of neurodegenerative diseases (Iturria-Medina and Evans, 2015), offering an overview on how connectivity dysfunctions mediate neurodegeneration, with a specific focus on how these dysfunctions are related to normal aging and the progression of neuropathologic changes.

This review article links with the original research article by Yi et al. (2015) who showed that differences in functional brain connectivity alterations are associated with cerebral amyloid deposition in a-MCI. Compared to controls, non-amnestic MCI showed atrophy in bilateral superior temporal gyri whereas a-MCI showed atrophy in right precuneus. The results indicate that “despite the similarity in cross-sectional cognitive features, non-amnestic MCI has quite different functional brain connectivity compared to a-MCI.”

More on brain connectivity; researchers from China used a resting-state fMRI approach to compare connectivity in healthy controls with connectivity in PD with tremor (Zhang et al., 2015). Patients showed increased centrality in the occipital, parietal and frontal regions while decreased centrality in the thalamus and the cerebellum anterior lobe. Seeded at these regions, a distributed network was further identified that encompassed cortical and subcortical regions, as well as the brainstem and the cerebellum. Graph-based analyses of this network revealed “increased information transformation efficiency in the group of patients.” Moreover, the identified network correlated with patients' clinical manifestations and this finding could distinguish controls from patients. Together, these results “provide a comprehensive view of network disorganization in PD with tremor and have important implications for understanding neural substrates underlying this specific type of PD.”

From imaging markers to biochemistry markers of PD: researcher found elevated levels of cerebrospinal fluid α-synuclein oligomers in healthy asymptomatic LRRK2 mutation carriers (Aasly et al., 2014). An inverse correlation between disease severity and duration and CSF levels of α- synuclein oligomers was observed. This study suggests that quantification of α-synuclein oligomers in CSF has potential value as a tool for PD diagnosis and presymptomatic screening of high-risk individuals.

PD is traditionally regarded as a neurodegenerative movement disorder, however, nigrostriatal dopaminergic degeneration is also thought to disrupt non-motor loops connecting basal ganglia to areas in frontal cortex involved in cognition and emotion processing. A couple of studies focused on the interface between motor, neuropsychological and neuropsychiatric features of PD. Researchers from Switzerland showed apathy in PD is related to executive function, gender and age but not to depression. Authors conclude that initiation dysfunction heralds apathy in PD. Even more, apathy is influenced by age and gender: older age correlates with apathy in men, whereas in women it seems to protect against it (Meyer et al., 2015).

In an interventional study, researchers from Nashville and Louisville (USA), assessed emotion recognition in early PD (EPD) undergoing deep brain stimulation or dopaminergic therapy, compared with healthy controls (McIntosh et al., 2015). EPD patients were impaired on all emotion recognition tasks. Neither therapy type nor therapy state (ON/OFF) altered emotion recognition performance. Finally, elderly controls were impaired on vocal emotion recognition relative to young controls, suggesting a physiological decline related to normal aging. In conclusion, emotion recognition is impaired early in PD, implicating the disruption of fronto-striatal loops mediating emotional function is an early phenomenon in PD.

Other interventional study addressed the effects of combined therapy (MAO-B inhibitors with levodopa) vs. monotherapy in PD (Krishna et al., 2014). Authors found that combined MAO-I and levodopa improves cognition compared to monotherapy. MAO-I combined with levodopa improves neuropsychiatric measures such as depression, apathy, anxiety as well as quality of life. This enhancing effect of combined therapy was more pronounced in PD patients with severe akinesia, compared to patients with severe tremor, suggesting akinetic patients particularly benefit from combined therapy.

A third interventional study, this time from France, assessed neuropsychiatric fluctuations in PD in ON and in OFF and compared them to controls (Fleury et al., 2014). The Visual Analog Mood (VAMS) and the Apathy scores improved by the acute intake of levodopa. Negative emotional Stroop task induced a lengthening of the mean reaction time during the incongruent trials compared with the congruent trials in controls and in ON patients, but not in OFF patients. OFF patients showed lower activation than Controls and ON patients within the right pregenual anterior cingulate cortex (pACC), an area specifically involved in emotional conflict resolution. Thus, pACC hypoactivation may contribute to explain neuropsychiatric fluctuations in PD. Emotional conflict processes should be understood as dopamine-dependent, therefore the practical learning point is that adjustments of dopaminergic medication might be helpful for treating these non-motor symptoms.

To summarize, this Research Topic is plenty of interesting articles from different and mutually enriching perspectives and methodologies, all around neuropsychological and neuropsychiatric aspects of neurodegenerative diseases. For us, it has been a pleasure serving as editors of these publications. We have learned both from authors and reviewers in the process of improving the original manuscripts until the final version of these papers was ready to be published. We hope readers also find this collection of articles interesting. Enjoy!

## Conflict of interest statement

The authors declare that the research was conducted in the absence of any commercial or financial relationships that could be construed as a potential conflict of interest.
